# A multi-center phase II study of high dose interleukin-2 sequenced with vemurafenib in patients with BRAF-V600 mutation positive metastatic melanoma

**DOI:** 10.1186/s40425-018-0387-x

**Published:** 2018-07-27

**Authors:** Joseph I. Clark, Jatinder Singh, Marc S. Ernstoff, Christopher D. Lao, Lawrence E. Flaherty, Theodore F. Logan, Brendan Curti, Sanjiv S. Agarwala, Bret Taback, Lee Cranmer, Jose Lutzky, Theresa L. Luna, Sandra Aung, David H. Lawson

**Affiliations:** 1 Cardinal Bernardin Cancer Center, Loyola University Medical Center, 2160 S. First Avenue, Maywood, IL 60153 USA; 2 Primary Biostatistical Solutions, Victoria, BC Canada; 3 Roswell Park Cancer Institute, Buffalo, NY USA; 4 University of Michigan, Ann Arbor, MI USA; 5 The Karmanos Cancer Institute, Detroit, MI USA; 6 Indiana University, Indianapolis, IN USA; 7 Earle A. Chiles Research Institute, Providence Cancer Center, Portland, OR USA; 8 St. Luke’s Hospital and Health Network, Bethlehem, PA USA; 9 Columbia University/Herbert Irving Comprehensive Cancer Center, New York, NY USA; 10 Fred Hutchinson Cancer Research Center, University of Washington, Seattle, WA USA; 11 Mt. Sinai Comprehensive Cancer Center, Miami Beach, FL USA; 12 Prometheus Laboratories Inc, San Diego, CA USA; 13 Emory Winship Cancer Institute at Emory University, Atlanta, GA USA; 14 Nektar Inc, San Diego, CA USA

**Keywords:** High-dose interleukin-2, Vemurafenib, BRAF-mutated metastatic melanoma, Multicenter, Phase II

## Abstract

**Background:**

Preclinical studies suggest that BRAF inhibitors enhance anti-tumor immunity and antigen presentation. Combination BRAF inhibition with immunotherapy is an appealing therapeutic approach. We sequenced vemurafenib with HD IL-2 in patients with BRAF-mutated metastatic melanoma to improve long term outcomes.

**Methods:**

Eligible patients were HD IL-2 eligible with metastatic BRAF V600 mutated melanoma. Cohort 1 was treatment naïve and received vemurafenib 960 mg BID for 6 weeks before HD IL-2. Cohort 2 received vemurafenib for 7–18 weeks before enrollment. Both cohorts received HD IL-2 at 600,000 IU/kg every 8 h days 1–5 and days 15–19. The primary objective was to assess complete responses (CR) at 10 weeks ±3 (assessment 1) and 26 weeks ±3 (assessment 2) from the start of HD IL-2.

**Results:**

Fifty-three patients were enrolled, (cohort 1, *n* = 38; cohort 2, *n* = 15). Of these, 39 underwent assessment 1 and 15 assessment 2. The CR rate at assessment 1 was 10% (95% CI 3–24) for both cohorts combined, and 27% (95% CI 8–55) at assessment 2. Three-year survival was 30 and 27% for cohort 1 and cohort 2, respectively. No unexpected toxicities occurred. A shift in the melanoma treatment landscape during this trial adversely affected accrual, leading to early trial closure.

**Conclusions:**

Vemurafenib in sequence with HD IL-2 did not change the known toxicity profile for either agent. Lower than expected response rates to vemurafenib were observed. Overall response rates and durability of responses appear similar to that observed with HD IL-2 alone.

**Trial registration:**

NCTN, NCT01683188. Registered 11 September 2012, http://www.clinicaltrials.gov/NCT01683188

## Background

Treatment of metastatic malignant melanoma has dramatically advanced with the development of targeted agents and immunotherapy in the recent past. Targeting the MAPK pathway with BRAF inhibitors, MEK inhibitors, or the combination, leads to high response rates and progression free survivals of roughly 7 months for single agents [1–3], to about 11 months for combination regimens [4, 5]. Approved immunotherapies now include high dose interleukin-2 (HD IL-2) and the immune checkpoint inhibitors, (ipilimumab, nivolumab, pembrolizumab). Durable responses are consistently observed in a small percentage of patients with metastatic melanoma treated with HD IL-2 [6–8]. Higher response rates are observed with immune checkpoint inhibitors [9–13], especially with combination anti-CTLA and anti-PD1 agents [14–16], but longer follow up is required to determine the durability of these responses and more recent studies report acquired resistance to these agents [17].

Preclinical studies suggest that oncogenic BRAF (BRAF V600E) may contribute to immune escape in melanoma [18], and that blocking its activity via MAPK inhibition leads to increased expression of melanocytic differentiation antigens (MDAs) [19, 20] with significantly enhanced recognition by antigen-specific T lymphocytes [20, 21], enhanced antigen presentation [22, 23], without diminishing T-cell function [24], and change in the tumor-produced immune environment [25]. The combination of BRAF inhibition with systemic immunotherapy is therefore an appealing therapeutic approach in the treatment of patients with advanced melanoma. That is, the effects of BRAF inhibition, including disease reduction and control for several months may enhance the likelihood of complete response to HD IL-2 therapy with subsequent anticipated improved durability of these responses.

In this open-label phase 2 trial we sequenced the BRAF inhibitor vemurafenib with HD IL-2 in the treatment of patients with stage IV, metastatic BRAF-mutated malignant melanoma and assessed the toxicity and efficacy specifically with regard to the complete response rate from this combination.

## Methods

### Patients

Eligible patients were age ≥ 18 years with histologically confirmed, not surgically resectable due to extent of disease, measurable stage IV BRAF V600E- or V600 K-mutated malignant melanoma. Patients had to meet requirements for HD IL-2 therapy as previously described [6] and vemurafenib therapy per institutional guidelines. Treatment naïve patients were enrolled in Cohort 1; patients who had been receiving active treatment with vemurafenib for 7 to 18 weeks were enrolled in Cohort 2. Patients were excluded if they had received prior treatment with HD IL-2, ipilimumab or other highly selective BRAF, MEK, NRAS, or cMET inhibitors. Prior treatment with an anti-PD-1 or anti-PDL-1 antibody was allowed. Exclusion also included a prolonged QTc interval of > 500 ms; known or suspected infection with HIV, hepatitis C, hepatitis B or other infectious hepatitis; pregnant or nursing women; untreated brain metastases; prior investigational drug within 30 days. Human investigations were performed after approval by an institutional review board or ethics committee at each participating institution and in accordance with an assurance filed with and approved by the U.S. Department of Health and Human Services. Written informed consent was obtained on all patients.

### Study design and treatment

**Fig. 1:**
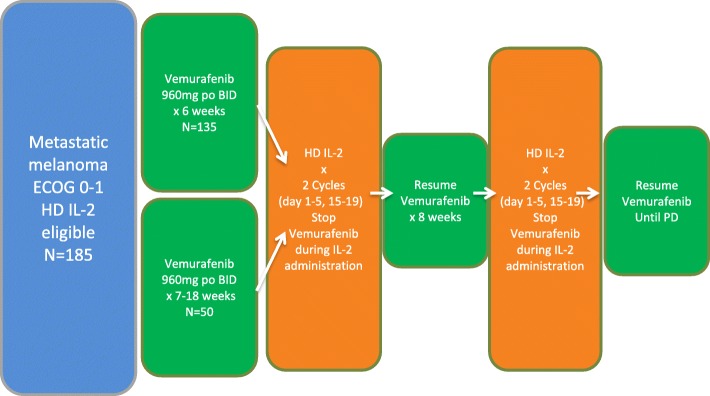
Treatment Schema

The study was approved by the institutional review board or ethics committee at each participating institution. Eligible patients who were treatment naïve were enrolled in Cohort 1 and received vemurafenib 960 mg by mouth twice daily for 6 weeks prior to receiving inpatient HD IL-2. Cohort 2 patients included eligible patients who had been receiving vemurafenib therapy for 7 to 18 weeks with stable or responding disease prior to enrollment. Baseline imaging studies were performed prior to starting vemurafenib in Cohort 1 and prior to starting HD IL-2 in Cohort 2. Both cohorts received HD IL-2 at 600,000 IU/kg intravenously over 15 min every 8 h for a maximum of 14 doses days 1–5, (cycle 1), and days 15–19, (cycle 2). Each course consisted of 2 cycles. Vemurafenib was held during inpatient HD IL-2 therapy, (Fig. 1), and resumed during the 9 days between cycles and at the completion of cycle 2. Dose adjustments of vemurafenib or withholding of HD IL-2 doses were performed by the respective treating physicians based on accepted standards of care for each of these agents. QTc intervals were reviewed daily for changes during each 5 day cycle of HD IL-2 dosing.

### Study assessments/statistical analysis

Baseline imaging for cohort 1 occurred within 4 weeks of starting vemurafenib whereas for cohort 2, baseline imaging occurred after patients had been receiving vemurafenib for > 7 weeks but < 18 weeks and either an objective response or stable disease had to have been observed before enrollment and initiation of HD IL-2 therapy. Disease response assessment occurred at week 10 ± 3 weeks, (assessment 1), and at week 26 ± 3 weeks, (assessment 2), from the start of HD IL-2 dosing. Patients received up to 2 courses of HD IL-2 if evidence of disease response was observed after the first course. Course 2 began at least 8 weeks after the completion of course 1. RECIST and immune-related response criteria (irRC) were used to assess response [26].

Cohort 1 was used to define the study size and statistical validity with the comparator being historical controls using data from BRAF positive patients from the Melanoma Select study (NCT01288963) [27]. The initial design was to enroll 135 subjects in Cohort 1. Based on a one-sample binomial test (using normal approximation), a sample size of 135 would have 80% power to detect a significant difference using a one-sided test with α = 0.05% if the true complete response (CR) rate for naïve subjects treated with 6 weeks of vemurafenib prior to adding HD IL-2 was twice the CR rate of the historical control for HD IL-2 (12% vs 6%). Up to 50 subjects were allowed to be enrolled in Cohort 2. This cohort was designed to evaluate whether additive or synergistic clinical benefit or toxicity was observed in BRAF-mutated melanoma patients treated with vemurafenib as a single agent for > 7 to 18 weeks prior to the first course of HD IL-2 therapy in conjunction with continued vemurafenib.

Secondary endpoints included (1) assessment of tumor response in patients with CR or near CR (> 90%) after discontinuation of vemurafenib, (2) compare progression free survival (PFS) from initiation of vemurafenib between Cohort 1 and Cohort 2 patients, (3) determine the duration of response in patients treated with vemurafenib and HD IL-2, (4) compare overall PFS with the historical data using vemurafenib or HD IL-2 alone, (5) compare safety between Cohort 1 and Cohort 2 patients, (6) compare safety between patients treated with vemurafenib and HD IL-2 versus historical HD IL-2 alone, (7) explore potential biomarkers for their predictive value in combination therapy of vemurafenib and HD IL-2, (8) assess treatment response to retreatment with vemurafenib in “CR” patients progressing on no therapy.

## Results

A total of 53 patients with BRAF V600-mutated metastatic melanoma were enrolled onto this trial. Cohort 1 included 38 patients and cohort 2, 15 patients, none of whom received prior anti-PD1 or anti-PDL1 therapy (Table 1). All evaluable patients received vemurafenib and at least one dose of HD IL-2. Of the 53 patients enrolled, 39 completed assessment 1 and 15 completed assessment 2, respectively. A caveat to this trial required that patients be enrolled into the PROCLAIM registry [27] once they started HD IL-2 therapy, for purposes of capturing survival data. Fourteen patients’ data were not captured for assessment 1 for various reasons: seven patients never proceeded on to HD IL-2 treatment, thus they were not entered into the PROCLAIM registry, and as such their response information was not captured. Of these seven, two patients experienced an adverse event on vemurafenib and the decision was made not to proceed with HD IL-2 therapy; one patient withdrew consent before receiving HD IL-2; two patients were unable to continue on protocol treatment due to insurance issues; one patient experienced progression of disease while taking vemurafenib thus did not proceed onto HD IL-2 treatment; one patient chose not to proceed with HD IL-2 treatment. Of the other seven patients who did not undergo assessment 1: in two patients support for the trial terminated prior to assessment 1 and they were treated off study; four patients withdrew consent after starting HD IL-2; one patient experienced early disease progression shortly after starting HD IL-2 therapy.

**Table 1:** Patient Demographics

Characteristic	Cohort 1	Cohort 2
	Vemurafenib 6 weeks (*N* = 38)	Vemurafenib 7–18 weeks (*N* = 15)
Age (yr)		
Mean (SD)	48.6 (12.79)	48.5 (13.90)
Median	50.5	48.0
Range	21–73	26–67
Gender – no. (%)		
Male	15 (39.5)	6 (40.0)
Female	23 (60.5)	9 (60.0)
Race – no. (%)		
White	35 (92.1)	15 (100.0)
Decline	2 (5.3)	0 (0.0)
Other	1 (2.6)	0 (0.0)
Metastasis stage – no. (%)		
M1a	9 (23.7)	2 (13.3)
M1b	13 (34.2)	3 (20.0)
M1c	16 (42.1)	10 (66.7)
LDH level – no. (%)		
≤ ULN	17 (44.7)	5 (33.3)
> ULN	17 (44.7)	8 (53.3)
Not measured	4 (10.5)	2 (13.3)
Prior therapy – no. (%)		
Surgery	34 (89.5)	13 (86.7)
Radiation	7 (18.4)	8 (53.3)
Chemotherapy	4 (10.5)	1 (6.7)
Anti-PD1/PDL1	0	0
Mutation – no. (%)		
BRAF	38 (100.0)	15 (100.0)
cKIT	1 (2.6)	0 (0.0)
Other	1 (2.6)	0 (0.0)
Performance Status		
0	24 (63.2)	8 (53.3)
1	14 (36.8)	7 (46.7)

Race of ‘Other’ had the description ‘Not specified’ recorded

**Fig. 2:**
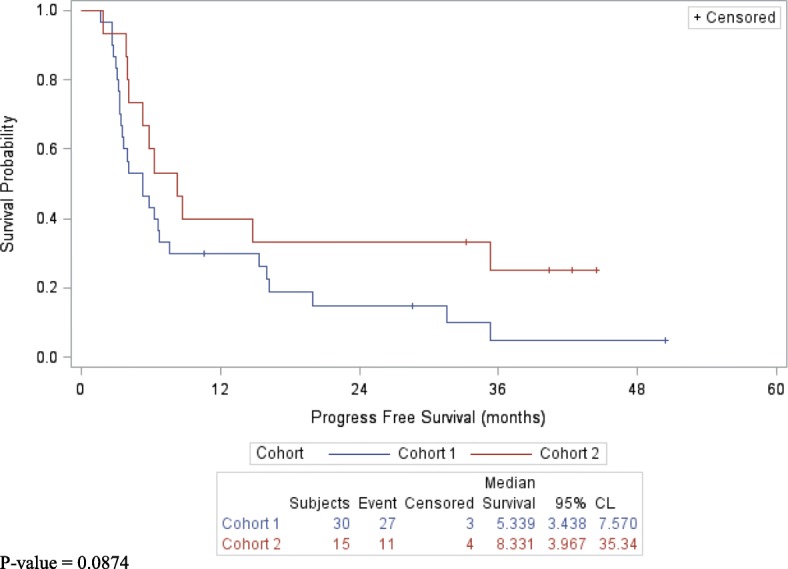
Progression-Free Survival by Cohort

**Fig. 3:**
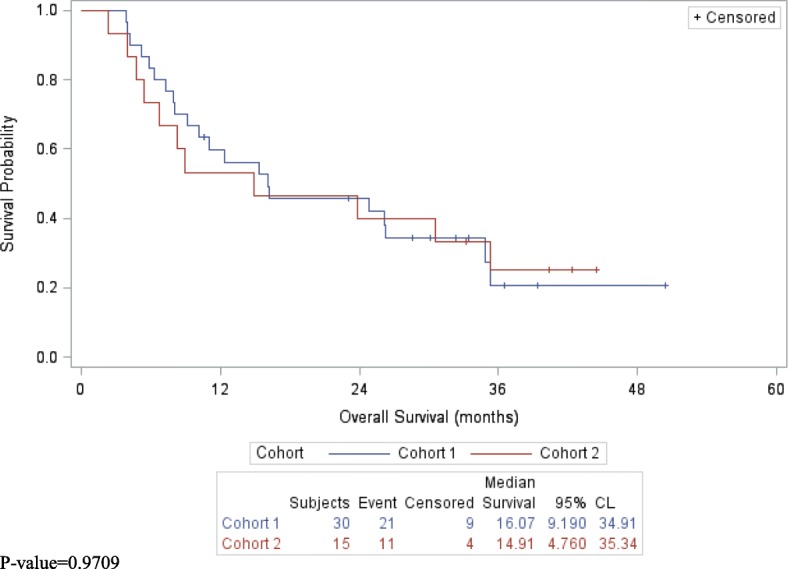
Overall Survival by Cohort

No difference between RECIST and irRC was observed. The best overall response rate, (ORR) (CR/PR), at assessment 1 was 27% (95% CI 12–46) and disease control rate, (CR/PR/SD) was 77% (95% CI 58–90) for cohort 1, (*n* = 30), and 27% (95% CI 8–55) and 73% (95% CI 45–92) for cohort 2 (*n* = 15), respectively, (Table 2). The CR rate was 10% (95% CI 2–17) for cohort 1 and 7% (95% CI 0.2–32) for cohort 2. At assessment 2, of the 15 patients who were assessed in both cohorts, the ORR was 60% (95% CI 33–84) and the disease control rate was 100% (95% CI 78–100). The median PFS was 5.3 months (95% CI 3.4–7.5) for cohort 1 and 8.3 months (95% CI 4.0–35.3) for cohort 2, (*p*-value = 0.0874), (Fig. 2). The duration of response ranged from 0.27 to 17+ months for all responders, and for those with stable disease up to 18.1+ months (Table 3). The median OS was 16.1 months (95% CI, 9.2–34.9) for cohort 1 and 14.9 months (95% CI 4.8–35.3) for cohort 2, (p-value = 0.9709), (Fig. 3). The 1, 2 and 3-year survivals were similar in both cohorts, (Table 4). No subjects who achieved a CR recurred.

**Table 2:** Best Overall Response

Group	Cohort 1 (*N* = 30)	Cohort 2 (*N* = 15)	Assessment 1 (*N* = 39)	Assessment 2 (*N* = 15)
Complete Response	3 (10%)	1 (7%)	4 (10%)	4 (27%)
95% CI (%)	(2, 27)	(0.2, 32)	(3, 24)	(8, 55)
Response (CR/PR)	8 (27%)	4 (27%)	11 (28%)	9 (60%)
95% CI (%)	(12, 46)	(8, 55)	(15, 45)	(33, 84)
Disease Control (CR/PR/SD)	23 (77%)	11 (73%)	30 (77%)	15 (100%)
95% CI (%)	(58, 90)	(45, 92)	(61, 89)	(78, 100)

**Table 3:** Duration of Response (Days)

Response		Cohort 1	Cohort 2
Complete Response	Mean (SD)	84 (93.8)	–
	Median	56	–
	Range	8–189	(ongoing)
Partial Response	Mean (SD)	194 (180.0)	113 (103.0)
	Median	118	133
	Range	77–512	1–204
Stable Disease	Mean (SD)	125 (156.8)	127 (186.3)
	Median	56	63
	Range	1–509	1–544

**Table 4:** 1-, 2-, 3-year Survival

Survival	Cohort 1 (*N* = 30)	Cohort 2 (*N* = 15)
1-year	18 (60%)	8 (53%)
2-year	14 (46.7%)	6 (40%)
3-year	9 (30%)	4 (26.7%)

No unexpected toxicities were observed with either vemurafenib or with HD IL-2. One patient in each cohort experienced a grade 3 rash with vemurafenib. Typical HD IL2-related toxicities including constitutional symptoms and capillary leak syndrome were observed in both cohorts without excess cardiac, hepatic, pulmonary, renal or skin toxicities beyond that expected with this agent. No prolongation of QT interval was observed in patients treated with HD IL-2 in either cohort. There were no treatment related deaths.

Although our intent was to pursue predictive biomarker data, due to funding issues and early closure this endeavor was aborted. A shift in the melanoma treatment landscape during this trial adversely affected accrual, in that combination BRAF/MEK inhibitor therapy soon became an approved and common standard treatment approach, while durable responses were achieved in a substantial percentage of patients treated with checkpoint inhibitors, which led to early trial closure.

## Discussion

Treatment options for advanced malignant melanoma have expanded significantly in the past number of years. When this trial was first conceptualized, in early 2011, those options were limited. HD IL-2 was the first systemic immunotherapy approved for patients with advanced melanoma but efficacy was observed in a select number of patients. Dramatic responses to the BRAF inhibitor, vemurafenib, and preclinical as well as early clinical reports of enhanced antigen expression and a favorable tumor microenvironment in patients treated with such targeted agents led to our attempt to combine this agent in BRAF-mutated melanoma [19–25]. Combination BRAF-MEK inhibitor therapy in this patient population was not yet approved when this trial initiated. Patient accrual to this study was negatively affected shortly after opening due to rapidly expanding treatment options for this group of patients, thus the trial closed to accrual prior to meeting its target population of 135 patients in Cohort 1.

The safety results of this study show vemurafenib combined with HD IL-2 did not change the known safety profile of either drug. No dose reductions were required for vemurafenib and there were no treatment related deaths. The overall response rate of approximately 27% for each cohort observed at assessment 1 was lower than expected for unclear reasons. One would expect a response rate of 40–50% for vemurafenib alone [1, 2], whereas response rates to HD IL-2 alone are consistently roughly 15–28% [6, 28, 29], although caution must be taken when comparing the current results to previously published data. Assessment 1 occurred at 10 ± 3 weeks from the start of HD IL-2. In Cohort 1, vemurafenib was taken for 6 weeks prior to starting HD IL-2, and in Cohort 2, vemurafenib was taken for 7 to 18 weeks prior to starting HD IL-2 but patients had to have had stable or responding disease before enrollment. Vemurafenib was held during each five-day cycle of inpatient HD IL-2 therapy in order to avoid excess toxicity especially hepatic or cardiac in nature. Systemic HD IL-2 may have adversely enhanced immunosuppression, e.g. via upregulating Treg function, which may help explain the dampening effect of vemurafenib therapy and may serve as one potential explanation for this lower than expected response rate. It is conceivable that if combination BRAF-MEK inhibitor therapy was used instead of single agent vemurafenib that observed response rates would have been higher. Biomarker assessment would enhance a better understanding of such an effect, but these assessments were not performed in this trial. Sullivan and colleagues have made an initial attempt at these evaluations in a similar smaller trial [30]. Disease control rate and 3-year overall survival, however, were as would be expected with HD IL-2 therapy alone in patients with advanced malignant melanoma [8, 27–29]. Thus this combination does not appear to induce and overall negative clinical outcome.

Combination targeted therapy with systemic immunotherapy is a valid approach worth investigation based on preclinical models and early clinical results [18–25, 31]. Caution must be heeded however as unexpected untoward toxicity may arise [32]. Active studies are ongoing using other BRAF/MEK combinations and immune checkpoint inhibitors, results of which are eagerly anticipated.

## Conclusions

In conclusion, results of this trial reveal that the combination of vemurafenib and HD IL-2 in the treatment of BRAF-mutated metastatic malignant melanoma was safe but without added clinical benefit beyond what would be expected with either agent alone. Accrual to the study was halted far short of planned due to a dramatic shift in the treatment landscape of this previously poorly responsive disease. Future alternative combination approaches remain worthy investigative endeavors.
